# An optimization formulation for characterization of pulsatile cortisol secretion

**DOI:** 10.3389/fnins.2015.00228

**Published:** 2015-08-11

**Authors:** Rose T. Faghih, Munther A. Dahleh, Emery N. Brown

**Affiliations:** ^1^Department of Electrical Engineering and Computer Science, Massachusetts Institute of TechnologyCambridge, MA, USA; ^2^Department of Brain and Cognitive Sciences, Massachusetts Institute of TechnologyCambridge, MA, USA; ^3^Department of Anesthesia, Critical Care and Pain Medicine, Massachusetts General HospitalBoston, MA, USA; ^4^Laboratory for Information and Decision Systems, Massachusetts Institute of TechnologyCambridge, MA, USA; ^5^Engineering Systems Division, Massachusetts Institute of TechnologyCambridge, MA, USA; ^6^Institute for Data, Systems, and Society, Massachusetts Institute of TechnologyCambridge, MA, USA; ^7^Institute for Medical Engineering and Science, Massachusetts Institute of TechnologyCambridge, MA, USA; ^8^Department of Anesthesia, Harvard Medical SchoolBoston, MA, USA

**Keywords:** pulsatile control, cortisol secretion, endocrine control, mathematical modeling, circadian rhythm

## Abstract

Cortisol is released to relay information to cells to regulate metabolism and reaction to stress and inflammation. In particular, cortisol is released in the form of pulsatile signals. This low-energy method of signaling seems to be more efficient than continuous signaling. We hypothesize that there is a controller in the anterior pituitary that leads to pulsatile release of cortisol, and propose a mathematical formulation for such controller, which leads to impulse control as opposed to continuous control. We postulate that this controller is minimizing the number of secretory events that result in cortisol secretion, which is a way of minimizing the energy required for cortisol secretion; this controller maintains the blood cortisol levels within a specific circadian range while complying with the first order dynamics underlying cortisol secretion. We use an ℓ_0_-norm cost function for this controller, and solve a reweighed ℓ_1_-norm minimization algorithm for obtaining the solution to this optimization problem. We use four examples to illustrate the performance of this approach: (i) a toy problem that achieves impulse control, (ii) two examples that achieve physiologically plausible pulsatile cortisol release, (iii) an example where the number of pulses is not within the physiologically plausible range for healthy subjects while the cortisol levels are within the desired range. This novel approach results in impulse control where the impulses and the obtained blood cortisol levels have a circadian rhythm and an ultradian rhythm that are in agreement with the known physiology of cortisol secretion. The proposed formulation is a first step in developing intermittent controllers for curing cortisol deficiency. This type of bio-inspired pulse controllers can be employed for designing non-continuous controllers in brain-machine interface design for neuroscience applications.

## 1. Introduction

Many hormones that have been well-investigated appear to be released in pulses (Stavreva et al., 2009); for example, cortisol, gonadal steroids, and insulin are released in a pulsatile manner (Veldhuis, 2008). Pulsatility is a physiological way of increasing hormone concentrations rapidly and sending distinct signaling information to target cells (Veldhuis, 2008). Ultradian pulsatile hormone secretion allows for encoding information via both amplitude and frequency modulation and is a way of frequency encoding (Lightman and Conway-Campbell, 2010; Walker et al., 2010b). Pulsatile signaling permits target receptor recovery, rapid changes in hormone concentration, and greater control, and is also more efficient than continuous signaling (Walker et al., 2010b). The mechanism underlying the generation of hormone pulses and why this method of signaling is chosen by the body over continuous signaling is not known. Since the transcriptional program prompted by hormone pulses is considerably different from constant hormone treatment (Stavreva et al., 2009), it is crucial to understand the physiology underlying pulsatile hormone release. Hormone pulsatility underlies multiple physiological processes. For example, (i) cortisol oscillations have crucial effects on target cell gene expression and glucocorticoids receptor function (McMaster et al., 2011; Walker et al., 2012). (ii) Some psychiatric and metabolic diseases are associated with changes in cortisol pulsatility (Walker et al., 2010a). (iii) When the same amount of corticosterone is administered by constant infusion rather than a pulsatile infusion, it results in a noticeably reduced ACTH response to stress (Lightman and Conway-Campbell, 2010). In this study, we investigate pulsatile release of cortisol and propose a novel mathematical formulation that characterizes pulsatile cortisol secretion.

Cortisol is released from the adrenal glands in pulses in response to pulsatile release of ACTH. CRH induces the release of ACTH. In return, cortisol has a negative feedback effect on ACTH and CRH release at the pituitary and hypothalamic levels. The timing and amplitudes of cortisol pulses vary throughout the day where the amplitude variations are due to the circadian rhythm underlying cortisol release with periods of 12 and 24 h (Faghih et al., 2011), and the variations in the timing of cortisol pulses result from the ultradian rhythm underlying cortisol release. Between 15 and 22 secretory pulses of cortisol are expected over 24 h (Veldhuis et al., 1989; Brown et al., 2001).

Based on the interactions in the HPA axis, it was hypothesized that pulsatile release of CRH from the hypothalamus results in pulsatile release of cortisol. Walker et al. suggest that a sub-hypothalamic pituitary-adrenal system results in the pulsatile ultradian pattern underlying cortisol release (Walker et al., 2012). This is because inducing constant CRH levels results in a pulsatile cortisol profile (Walker et al., 2012) while constant ACTH levels do not result in pulsatile cortisol secretion (Spiga et al., 2011). Spiga et al. suppressed the activity of the HPA axis by oral methylprednisolone and infused both constant amounts and pulses of ACTH to test the hypothesis that pulsatile ACTH release is necessary for pulsatile cortisol secretion (Spiga et al., 2011). While pulsatile ACTH resulted in pulsatile cortisol secretion, constant infusion of same amounts of ACTH did not activate cortisol secretion (Spiga et al., 2011). Moreover, studies on sheep in which the hypothalamus has been disconnected from the pituitary suggest that pulsatile input from hypothalamic secretagogues (e.g., CRH or vasopressin) is not necessary for the ultradian rhythm in cortisol secretion or for pulsatile cortisol secretion and pulsatile cortisol secretion is still maintained (Walker et al., 2010a). Hence, pulsatile cortisol release is controlled by the dynamics in the anterior pituitary. Since pulsatile cortisol release seems to be more efficient than continuous signaling, it might be the case that the anterior pituitary is solving an optimal control problem.

We postulate that there is a controller in the anterior pituitary that controls the pulsatile secretion of cortisol and the ultradian rhythm of the pulses via the negative feedback effect of cortisol on the anterior pituitary. Hence, by considering the known physiology of the HPA axis, we shall formulate an optimization problem that achieves impulse control. In optimal control theory, impulse control is a special case of bang-bang control, in which an action leads to instantaneous changes in the states of the system (Sethi and Thompson, 2000). Impulse control occurs when there is not an upper bound on the control variable and an infinite control is exerted on a state variable in order to cause a finite jump (Sethi and Thompson, 2000). Minimizing an ℓ_0_-norm cost function can achieve impulse control and we use a reweighed ℓ_1_-norm formulation as a relaxation to the ℓ_0_-norm to solve the proposed optimization formulation. Moreover, we consider the first-order dynamics underlying cortisol synthesis and the circadian amplitude constraints on the cortisol levels when formulating the optimization problem.

## 2. Methods

We propose a physiologically plausible optimization problem for cortisol secretion by making the following assumptions: (1) Cortisol levels can be described by first-order kinetics for cortisol synthesis in the adrenal glands, cortisol infusion to the blood, and cortisol clearance by the liver described in Brown et al. (2001), Faghih (2010), and Faghih et al. (2011, 2014). (2) There is a time-varying cortisol demand [*h*(*t*)] that should be satisfied throughout the day, which is a function of the circadian rhythm. (3) There is a time-varying upper bound on the cortisol level [*q*(*t*)] that is a function of the upper bound on the cortisol level that the body can produce or a holding cost so that the cortisol level would not be much above the demand. (4) Control that results in cortisol secretion [*u*(*t*)] is non-negative. (5) The body is minimizing the number of resources (control) throughout the day. Hence, we postulate that there is a controller in the anterior pituitary that controls cortisol secretion via the following optimization formulation:

(1) $$\underset{u}{\min}\;\|u{\|}_{0}$$

$$\begin{array}{l} \text{s}.\text{t}.\\ \;u(t)\geq 0\\ \;\frac{dx_{1}(t)}{dt}=-\lambda x_{1}(t)+u(t)\\ \;\frac{dx_{2}(t)}{dt}=\lambda x_{1}(t)-\gamma x_{2}(t)\\ \;h(t)\leq x_{2}(t)\leq q(t) \end{array}$$

where *x*_1_ is the cortisol concentration in the adrenal glands and *x*_2_ is the blood cortisol concentration. λ and γ, respectively, represent the infusion rate of cortisol from the adrenal glands into the blood and the clearance rate of cortisol by the liver.

Considering the known physiology of *de novo* cortisol synthesis (i.e., no cortisol is stored in the adrenal glands) (Brown et al., 2001), we assume that the initial condition of the cortisol level in the adrenal glands is zero [*x*_1_(0) = 0] (Brown et al., 2001). Assuming that the input and the states are constant over 1-min intervals, and *y*_0_ is the initial condition of the blood cortisol concentration, blood cortisol levels at every minute over *N* min can be represented in discrete form by $\text{y}={\left[\begin{array}{cccc} y_{1} & y_{2} & \cdots & y_{N} \end{array}\right]}^{\prime }$ where *y*_*k*_ is the blood cortisol level at time *k* and y can be represented as:

(2) $$\text{y}=Fy_{0}+G\text{u}$$

where $F={\left[\begin{array}{cccc} f_{1} & f_{2} & \cdots & f_{N} \end{array}\right]}^{\prime },f_{k}=e^{-\gamma k},G={\left[\begin{array}{cccc} g_{1} & g_{2} & \cdots & g_{N} \end{array}\right]}^{\prime }$, $g_{k}={\left[\begin{array}{ccc} \frac{\lambda }{\lambda -\gamma }(e^{-\gamma k}-e^{-\lambda k}) & ... & \frac{\lambda }{\lambda -\gamma }(e^{-\gamma }-e^{\lambda })\underset{N-k}{\underset{︸}{0\;\cdots \;0}} \end{array}\right]}^{\prime }$, and u represents the control over *N* min. Then by letting $\text{h}={\left[\begin{array}{cccc} h_{1} & h_{2} & \cdots & h_{N} \end{array}\right]}^{\prime }$ where *h*_*k*_ is the cortisol demand at an integer minute *k* and $\text{q}={\left[\begin{array}{cccc} q_{1} & q_{2} & \cdots & q_{N} \end{array}\right]}^{\prime }$ where *q*_*k*_ is the upper bound at the integer minute *k*. Hence, we solve the discrete analog of the formulation in Equation (1):

(3) $$\underset{\text{u},x_{0}}{\min}\|\text{u}{\|}_{0}$$

$$\begin{array}{l} \text{s}.\text{t}.\\ \text{ u}\geq 0\\ \text{ x}=Fy_{0}+G\text{u}\\ \text{ h}\leq \text{x}\leq \text{q} \end{array}$$

ℓ_0_ problems are generally NP-hard, and instead an ℓ_1_-norm relaxation of such problems can be solved. In solving ℓ_1_-norm problems, there is a dependence on the amplitude of the coefficients over which the ℓ_1_-norm is minimized, and there is more penalty on larger coefficients than on smaller ones. However, it is possible to strategically construct a reweighted ℓ_1_-norm such that non-zero coefficients are penalized in a way that the cost further resembles the ℓ_0_-norm. By putting large weights on small entries, the solution concentrates on entries with small weights, non-zero entries are discouraged in the recovered signal, and a cost function that is more similar to an ℓ_0_-norm cost function can be solved (Candes et al., 2008). To find such weights for ℓ_1_-norm cost function, Candes et al. (2008) have proposed an iterative algorithm for enhancing the sparsity using reweighted ℓ_1_ minimization, which solves $\underset{\text{u}}{\min}\|\text{u}{\|}_{0}$. This algorithm is based on Fazel's “log-det heuristic” algorithm for minimizing the number of non-zero entries of a vector (Fazel, 2002) and the convergence of this log-det heuristic algorithm has been studied in Lobo et al. (2007). Hence, we use the algorithm by Candes et al. (2008) such that the constraints in the optimization problem in Equation (3) are satisfied:

1. Initialize the diagonal matrix *W*^(0)^ with entries *w*^(0)^_*i*_ = 1, *i* = 1, …, *n* on the diagonal and zeros elsewhere
2. Solve ${\text{u}}^{\left(\ell \right)}=arg\underset{\text{u}}{\;\min}\|W^{\left(\ell \right)}\text{u}{\|}_{1}$ subject to the constraints in Equation (3)
3. Update the weights $w_{i}^{\left(\ell +1\right)}=\frac{1}{|{\text{u}}_{i}{}^{\left(\ell \right)}|+\epsilon }$, *i* = 1, …, *n*
4. Iterate till ℓ reaches a certain number of iterations. Otherwise, increment ℓ and go to step 2.

The idea is that by solving u(ℓ+1)= arg   minu∑i=1n|ui||ui(ℓ)|+ϵ iteratively, the algorithm attempts to solve for a local minimum of a concave penalty function that is more similar to the ℓ_0_-norm (Candes et al., 2008). ϵ is used to ensure that weights on the recovered zero entries will not be set to ∞ at the next step, which would prevent us from obtaining estimates at the next step. ϵ should be slightly larger than the expected non-zero amplitudes of the signal that is to be recovered, and a value of at least 0.001 is recommended (Candes et al., 2008). This algorithm does not always find the global minimum and as ϵ → 0, the likelihood of stagnating at an undesirable local minimum increases (Candes et al., 2008). For ϵ values closer to zero, the iterative reweighted ℓ_1_-norm algorithm stagnates at an undesirable local minimum (Candes et al., 2008).

We study the optimization problem in Equation (1) via four examples. We first investigate the case that the optimization formulation in Equation (1) is selecting the control such that the state (i.e., the blood cortisol concentration) is bounded between constant lower and upper bounds to illustrate the idea that the formulation in Equation (1) can achieve impulse control. Then, we investigate cases in which the upper and lower bounds have harmonic profiles with a circadian rhythm. Using the iterative algorithm for enhancing the sparsity by reweighted ℓ_1_ minimization (Candes et al., 2008), we solve the optimization problem in Equation (1) over a time period τ and update the solution after a time period $\frac{\tau }{2}$ and repeat this process for a 24-h period. λ, γ, ϵ, τ, and lower and upper bounds are given in Tables 1–**3**. Since empirically the algorithm converges in 10 iterations for the formulation in this study, we use ℓ = 10 when running the algorithm. Numerical analysis was performed in MATLAB R2011b and using CVX (Grant and Boyd, 2008, 2014).

**Table 1:** **Model parameters for examples of optimization problem (Equation 1)**.

**Example**	**λ(min^−1^)**	**γ(min^−1^)**	**$\epsilon (\frac{\text{ug}}{\text{dl.min}})$**	**τ (min)**
1	0.0585	0.0122	0.01	360
2	0.0585	0.0122	0.0055	360
3	0.0585	0.0122	0.0075	360
4	0.1248	0.0061	0.0075	360

*The parameters λ and γ are, respectively, the infusion rate of cortisol into the circulation from the adrenal glands and the clearance rate of cortisol by the liver, and were both obtained from Faghih et al. (2014). The parameter ϵ provides stability for the iterative algorithm for enhancing the sparsity by reweighted ℓ_1_ minimization (Candes et al., 2008), and τ is the period over which we solve the iterative algorithm*.

## 3. Results

To illustrate that the proposed approach results in impulse control, we use constant lower and upper bounds and show that the proposed method achieves impulse control and a state that has a pulsatile profile. This example is not physiological and is used to help the reader better understand the type of results this type of approach generates. Then, we show an example that corresponds to a healthy subject and leads to impulse control. The secretory events and cortisol levels are in agreement with physiologically plausible profiles in healthy human data, and the obtained solution is optimal. Moreover, we illustrate another example that corresponds to a healthy subject and achieves impulse control. In this example, while the secretory events and cortisol levels are physiologically plausible, the obtained solution is optimal over the first 20 h and suboptimal for the last 4 h. This example shows that the performance of the algorithm used for solving the proposed optimization formulation depends on the choice of ϵ and can stagnate at a local minimum. Finally, we provide an example that illustrates a case in which the number of pulses is not within a physiologically plausible range (i.e., an abnormality) while impulse control is achieved.

### 3.1. Example 1

**Figure 1:**
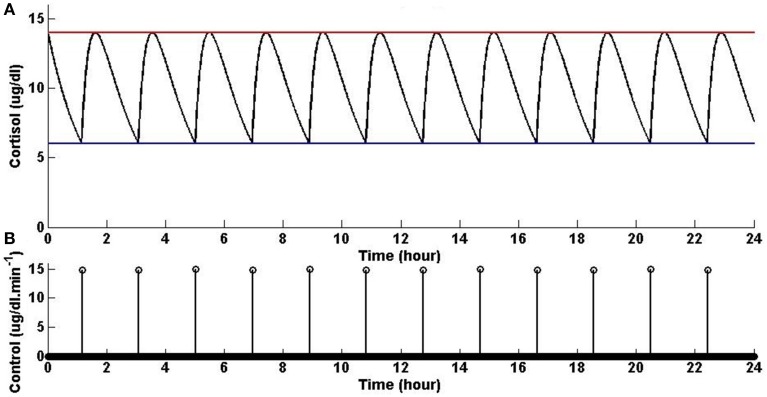
**Cortisol levels and control obtained using Example 1**. **(A)** The top panel displays the optimal cortisol profile (black curve), constant upper bound (red curve), and constant lower bound (blue curve). **(B)** The bottom panel displays the optimal control. The optimization problem obtained 12 impulses over 24 h as the optimal control (the timing of the control was discretized into 1440 points; the obtained control takes 12 non-zero values, i.e., impulses, while it is zero everywhere else). The optimization problem was solved using the parameters given in Example 1 in Table 1 and the upper and lower bounds provided in Tables 2, 3, respectively.

Assuming that the upper and lower bounds are constant, the optimal solution is achieved when the initial condition starts at the upper bound; then, when the state decays to the lower bound, an impulse causes a jump in the state which brings it back to the upper bound, and then again the state decays to the lower bound and the same jump to the upper bound again occurs, and the same process keeps repeating. Figure 1 shows that solving the optimization problem (Equation 1) for constant upper and lower bounds using the parameters given for Example 1 in Table 1 and the upper and lower bounds provided in Tables 2, 3, respectively, results in impulse control. There are 12 constant impulses obtained over a 24-h period, which occur periodically. This example is just a simple toy problem illustrating that the optimization formulation in Equation (1) can achieve impulse control and pulsatile cortisol release using a low energy input. This example does not have any physiological implications for cortisol secretion as it does not include upper and lower bounds that have a circadian rhythm observed in cortisol levels.

**Table 2:** **Upper bounds for examples of optimization problem (Equation 1)**.

**Example**	**$q(t)(\frac{\text{ug}}{}\text{dl})$**
1	14
2	$5.3782+0.3939\text{sin}(\frac{2\pi t}{1440})-3.5550\text{cos}(\frac{2\pi t}{1440})-0.5492\text{sin}(\frac{2\pi t}{720})+1.0148\text{cos}(\frac{2\pi t}{720})$
3	$8.6051+3.0306\text{sin}(\frac{2\pi t}{1440})-5.0931\text{cos}(\frac{2\pi t}{1440})-1.8151\text{sin}(\frac{2\pi t}{720})-1.6570\text{cos}(\frac{2\pi t}{720})$
4	$8.6051+3.0306\text{sin}(\frac{2\pi t}{1440})-5.0931\text{cos}(\frac{2\pi t}{1440})-1.8151\text{sin}(\frac{2\pi t}{720})-1.6570\text{cos}(\frac{2\pi t}{720})$

*q(t) is the upper bound on the cortisol level*.

**Table 3:** **Lower bounds for examples of optimization problem (Equation 1)**.

**Example**	**$h(t)(\frac{\text{ug}}{\text{dl}})$**
1	6
2	$3.2478-0.7813\text{sin}(\frac{2\pi t}{1440})-2.8144\text{cos}(\frac{2\pi t}{1440})-0.2927\text{sin}(\frac{2\pi t}{720})+1.3063\text{cos}(\frac{2\pi t}{720})$
3	$5.5065+1.5544\text{sin}(\frac{2\pi t}{1440})-4.3112\text{cos}(\frac{2\pi t}{1440})-1.6355\text{sin}(\frac{2\pi t}{720})-0.9565\text{cos}(\frac{2\pi t}{720})$
4	$5.5065+1.5544\text{sin}(\frac{2\pi t}{1440})-4.3112\text{cos}(\frac{2\pi t}{1440})-1.6355\text{sin}(\frac{2\pi t}{720})-0.9565\text{cos}(\frac{2\pi t}{720})$

*h(t) is the lower bounds on the cortisol level*.

### 3.2. Example 2

**Figure 2:**
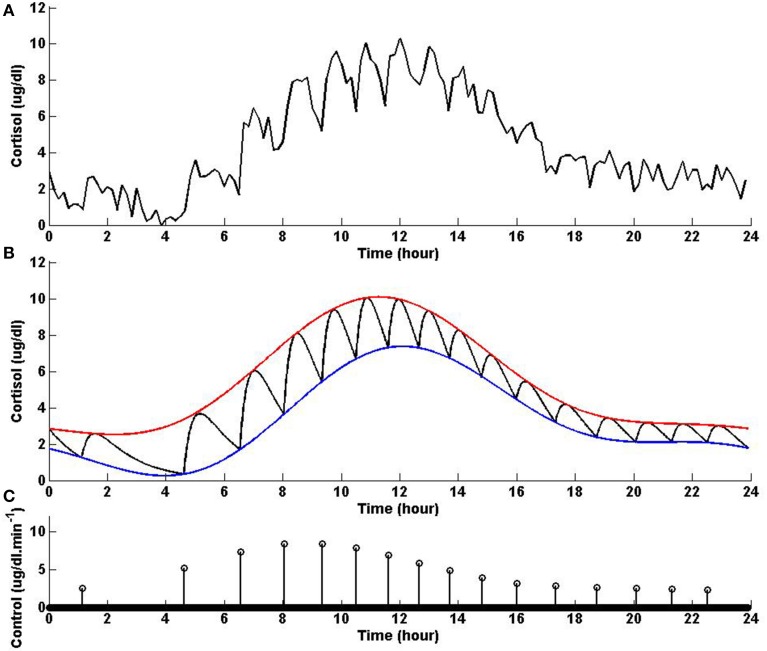
**Cortisol levels and control obtained using Example 2**. **(A)** The top panel displays the optimal cortisol profile obtained by adding a zero mean Gaussian measurement error with a standard deviation of σ = 0.45 to each simulated data point; the cortisol levels are recorded every 10 min. **(B)** The middle panel displays the optimal cortisol profile (black curve), two-harmonic upper bound (red curve), and two-harmonic lower bound (blue curve); the cortisol levels are recorded every minute. **(C)** The bottom panel displays the optimal control. The optimization problem obtained 16 impulses over 24 h as the optimal control (the timing of the control was discretized into 1440 points; the obtained control takes 16 non-zero values, i.e., impulses, while it is zero everywhere else). The optimization problem was solved using the parameters given in Example 2 in Table 1 and the upper and lower bounds provided in Tables 2, 3, respectively.

In healthy humans, cortisol levels have regular periodic time-varying patterns that consist of episodic release of secretory events with varying timings and amplitudes in a regular diurnal pattern. Figure 2 shows that solving the optimization problem (Equation 1) for two-harmonic bounds with a circadian rhythm, using the parameters given for Example 2 in Table 1 and the upper and lower bounds provided in Tables 2, 3, respectively, the obtained control is impulse control. Figure 2 also displays that adding a zero mean Gaussian measurement error with a standard deviation of σ = 0.45 to each simulated data point and recording the cortisol levels every 10 min (which is comparable to measurement noise and sampling interval of cortisol data in human subjects, Faghih et al., 2014), the obtained cortisol profile resembles cortisol human data provided in Faghih et al. (2014). There are 16 impulses over a 24-h period with time-varying circadian amplitudes and ultradian timings; the obtained control is within the physiologically plausible range of 15 –22 pulses (Veldhuis et al., 1989; Brown et al., 2001). The impulses are more frequent during the day and have higher amplitudes during the day than in night time. Obtained cortisol levels are low at night. Then, around 6 AM, cortisol levels increase, reaching higher values between 10 AM and 12 PM, followed by a gradual decrease throughout the day reaching low values at night. The obtained control and state are optimal; the state starts at the upper bound and decays to the lower bound at which point an impulse causes a jump in the system that results in increasing the state, and the state reaches the upper bound. Then, the state decays again to the time-varying lower bound and this process repeats. This example illustrates that the optimization formulation in Equation (1) can achieve impulse control and pulsatile cortisol release, using a low energy input, and generate secretory events and cortisol levels that have physiologically plausible profiles similar to those observed in healthy human data.

### 3.3. Example 3

**Figure 3:**
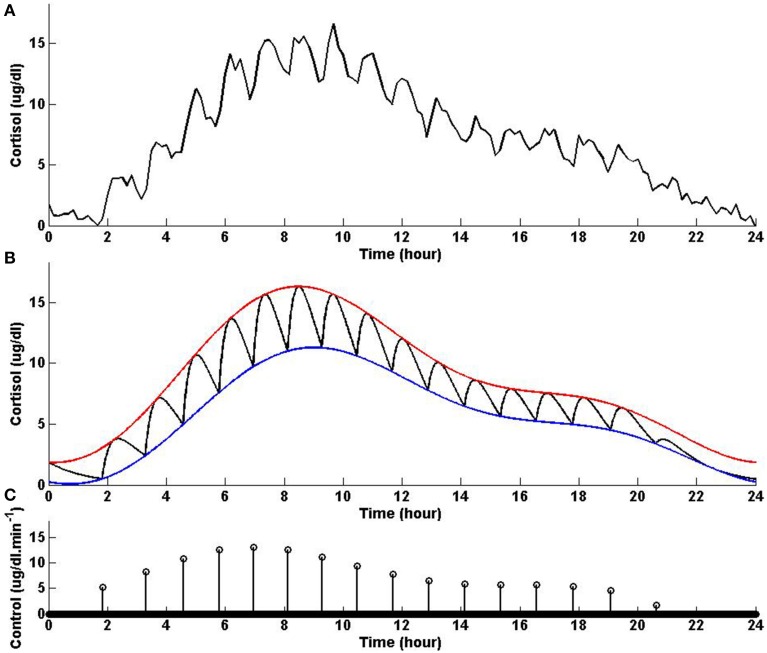
**Cortisol levels and control obtained using Example 3**. **(A)** The top panel displays the cortisol profile obtained by adding a zero mean Gaussian measurement error with a standard deviation of σ = 0.45 to each simulated data point; the cortisol levels are recorded every 10 min. **(B)** The middle panel displays the obtained cortisol profile (black curve), two-harmonic upper bound (red curve), and two-harmonic lower bound (blue curve). **(C)** The bottom panel displays the obtained control. The optimization problem obtained 16 impulses over 24 h as the control (the timing of the control was discretized into 1440 points; the obtained control takes 16 non-zero values, i.e., impulses, while it is zero everywhere else). The optimization problem was solved using the parameters given in Example 3 in Table 1 and the upper and lower bounds provided in Tables 2, 3, respectively.

In this example, we consider different lower and upper bounds compared to Example 2 while keeping λ and γ to values used in Example 2. Figure 3 shows that solving the optimization problem (Equation 1) for two-harmonic bounds with a circadian rhythm, using the parameters given for Example 3 in Table 1 and the upper and lower bounds provided in Tables 2, 3, respectively, the obtained control is impulse control. Figure 3 also displays that adding a zero mean Gaussian measurement error with a standard deviation of σ = 0.45 to each simulated data point and recording the cortisol levels every 10 min (which is comparable to measurement noise and sampling interval of cortisol data in human subjects, Faghih et al., 2014), the obtained cortisol profile resembles cortisol human data provided in Faghih et al. (2014). Sixteen impulses are obtained over 24 h which is within the physiological range of 15–22; these impulses have time-varying circadian amplitudes and ultradian timings. The impulses have higher amplitudes and are more frequent between 4 AM and 12 PM. The obtained cortisol levels are low at night. Then, the cortisol levels increase, reaching higher values between 7 AM and 11 AM, followed by a gradual decrease throughout the day, reaching low values at night. This example illustrates that the optimization formulation in Equation (1) can achieve impulse control and pulsatile cortisol release using a low energy input, and generates secretory events and cortisol levels that have physiologically plausible profiles similar to those observed in healthy human data. The control and state obtained in the first 20 h are optimal; however, the control and the state obtained for the last 4 h are suboptimal as the algorithm used for solving the optimization problem (Equation 1) can stagnate at a local minimum depending on the choice of ϵ. However, still a low energy control is recovered that keeps the cortisol levels within the desired bounds.

### 3.4. Example 4

**Figure 4:**
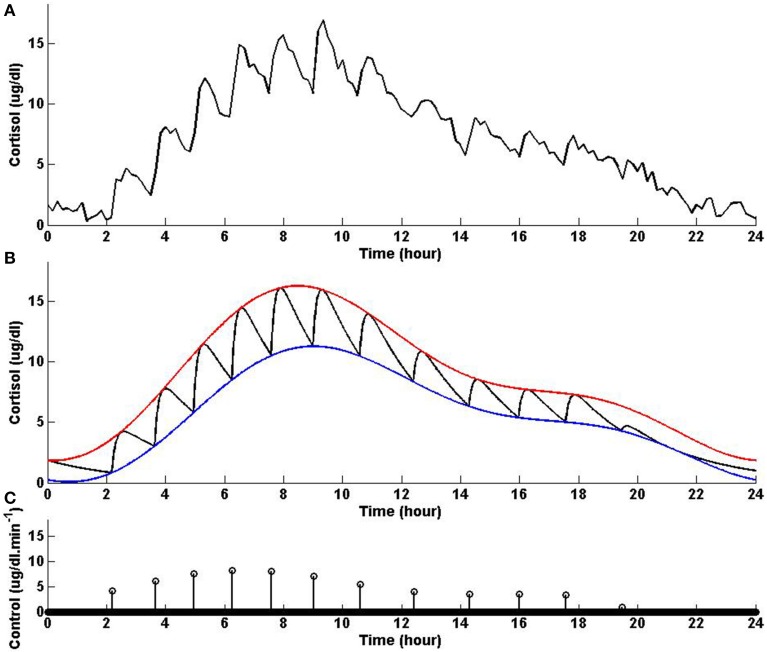
**Cortisol levels and control obtained using Example 4**. **(A)** The top panel displays the cortisol profile obtained by adding a zero mean Gaussian measurement error with a standard deviation of σ = 0.45 to each simulated data point; the cortisol levels are recorded every 10 min. **(B)** The middle panel displays the obtained cortisol profile (black curve), two-harmonic upper bound (red curve), and two-harmonic lower bound (blue curve). **(C)** The bottom panel displays the obtained control. The optimization problem obtained 12 impulses over 24 h as the control (the timing of the control was discretized into 1440 points; the obtained control takes 12 non-zero values, i.e., impulses, while it is zero everywhere else). The optimization problem was solved using the parameters given in Example 4 in Table 1 and the upper and lower bounds provided in Tables 2, 3, respectively.

In this example, we keep the lower and upper bounds the same as the values we used in Example 3 while using values for λ and γ that result in higher infusion of cortisol and lower clearance of cortisol compared to Example 3. Figure 4 shows that solving the optimization problem (Equation 1) using the parameters given for Example 4 in Table 1 and the upper and lower bounds provided in Tables 2, 3, respectively, the obtained control is impulse control. Figure 4 also displays that adding a zero mean Gaussian measurement error with a standard deviation of σ = 0.45 to each simulated data point and recording the cortisol levels every 10 min (which is comparable to measurement noise and sampling interval of cortisol data in human subjects Faghih et al., 2014), the obtained cortisol profile resembles cortisol human data provided in Faghih et al. (2014). Twelve impulses are obtained over 24 h where the impulses have lower amplitudes and are less frequent compared to the impulses obtained in Example 3. The obtained impulses still have time-varying circadian amplitudes and ultradian timings. The number of pulses has decreased compared to Example 3 which was expected as cortisol is cleared faster in this example. While the number of these pulses are not within the physiological range reported for healthy subjects, the obtained cortisol levels are still within the desired range. Cortisol levels are low at night, then increase, reaching higher values between 6 AM and 10 AM, followed by a gradual decrease throughout the day, reaching low values at night. The peak values of cortisol levels change and on average in this example the cortisol levels have lower values, and this might illustrate a case of cortisol deficiency. Also, in this example, the optimization formulation in Equation (1) results in impulse control and pulsatile cortisol release using a low energy input. The control and state obtained in the first 19 h are optimal; however, the control and the state obtained for the last 5 h are suboptimal as the algorithm used for solving the optimization problem (Equation 1) can stagnate at a local minimum depending on the choice of ϵ. However, still a low energy control is recovered that keeps the cortisol levels within the desired bounds.

## 4. Discussion

It is well-known that cortisol is released in pulses, and based on our results it appears that this method of relaying information might be an optimal approach as opposed to continuous signaling. In this work, we formalized this concept by proposing an optimization formulation for a physiologically plausible controller in the anterior pituitary that achieves impulse control as the optimal solution. In the proposed formulation, we assumed that there is a time-varying upper bound on the cortisol levels in the blood. Also, we assumed that the cortisol levels in the blood should be above a time-varying circadian threshold to achieve normal regulation of the HPA axis. We assumed that the lower bound and upper bound on the cortisol levels are two-harmonic functions with periods of 12 and 24 h that are controlled by the circadian rhythm. However, the upper bound and the lower bound for cortisol secretion could have multiple harmonics, and this assumption is only considering the most significant periods in cortisol release. Moreover, we considered the first-order dynamics underlying cortisol secretion. We have shown that the proposed optimization formulation yields impulse control as its optimal solution. The number, timing, and amplitude of the recovered secretory events in the proposed optimization problem are physiologically plausible. Moreover, the obtained cortisol profile is in agreement with the circadian rhythm observed in healthy human data. As pointed out, the iterative algorithm for enhancing the sparsity by reweighted ℓ_1_ minimization (Candes et al., 2008) does not always find the global minimum and might stagnate at an undesirable local minimum; we employed this algorithm to solve examples of optimization problems formulated in Equation (1) to show that the formulation in Equation (1) achieves impulse control as observed in cortisol levels. However, the optimization problem in Equation (1) can be solved using other methods as well, and for arbitrary choices of ϵ and τ the algorithm for enhancing the sparsity by reweighted ℓ_1_ minimization (Candes et al., 2008) might stagnate at a local minima and not achieve the optimal solution (please see Example 3).

To validate this mathematical characterization using experiments, one can start by recovering the parameters for a rat model and obtain lower and upper bounds on cortisol levels in a healthy rat. Next, make the adrenal glands of the rat malfunctional so that the rat becomes Addisonian and does not secrete cortisol. Then, using a pulse controller, obtain a cortisol profile that stays within the lower and upper bounds found when the rat was healthy.

While we proposed a simple optimization formulation that can achieve impulse control, it is possible to obtain impulse control using more complex formulations by either assuming that the system is a switched system with different rates or assuming that the nature of the system is impulsive and there is no continuous control. We assumed that the infusion and clearance rates are constant; however, the system can be a switched system with different infusion and clearance rates. Abrupt changes in the infusion and clearance rates could also result in impulse control. For example, if the infusion rate of cortisol from the adrenal glands starts from a constant level at wake and decreases abruptly to a new constant level, a very large level of cortisol should be produced in a short time so that the desired cortisol level can still be achieved. There could be multiple abrupt changes in the infusion rate throughout the day, and there might be an infusion rate reset to a high level at the beginning of sleep. Another example that could possibly result in impulse control is when the clearance starts at a constant level, and increases abruptly to a new constant level; then, a very large level of cortisol should be produced in a short time so that the desired cortisol level can still be achieved. There could be multiple such abrupt changes in the clearance rate throughout the day, and the clearance rate might be reset to a low level at the beginning of sleep. Another scenario could be that both the infusion and the clearance rates could be starting from a constant level and change abruptly to different levels periodically. In that case, the overall effect is that cortisol gets cleared faster or cortisol gets infused to the blood more slowly, and at such moments a very large cortisol level should be released for a short period of time to maintain the desired cortisol level. Such situations could possibly achieve impulse control as long as there is not an upper bound on the control variable; a mathematical example of a model with a time-varying rate that achieves impulse control is given in Sethi and Thompson (2000), and the *maximum principle* is used to find the optimality conditions for this problem. Moreover, it is possible that pulsatile inputs arise from the nature of the system, and the hormone system might be designed such that the input to the system can only be impulsive where the timing of the impulses are functions of the states and are not activated until a reseting condition is satisfied. A mathematical example of such a model is given in Wang and Balakrishnan (2008) where the cost function minimizes the energy in the input and the state, and calculus of variations is used to find the optimality conditions. Also, another possibility is that the body is solving a weighted ℓ_1_ cost function where different costs are associated with the control at different times of the day (e.g., the weights obtained at convergence when using the reweighted algorithm).

In this study, for modeling cortisol secretion, we proposed a physiologically plausible optimization formulation for a controller in the anterior pituitary. A similar approach can be used to study other endocrine hormones that are released in pulses. For example, the proposed optimization formulation can be tailored to include the constraints underlying thyroid hormone secretion or gonadal hormone secretion or growth hormone secretion in order to study the pulsatile release of those hormones. The transcriptional program stimulated by hormone pulses is very different from constant hormone treatment and some disorders are associated with hormone pulsatility. Hence, understanding the underlying nature of the pulsatile release of these hormones via mathematical formalization can be beneficial to understanding the pathological neuroendocrine states and treating some hormonal disorders.

In addition to contributing to the scientific advances in understanding cortisol regulation in daily rhythms, we provide a better understanding of the biological mechanism mathematically, which can potentially be used to come up with a similar approach to devise pulsatile control interventions instead of continuous controllers for treating cortisol disorders. Traditional control-theoretic methods do not normally consider the intermittent control that is observed in pulsatile control of cortisol release. Instead of developing a controller that tracks the desired cortisol levels, we have proposed a formulation for a controller that maintains the cortisol levels within certain upper and lower bounds. Our study formalizes, mathematically, the pulsatile controller underlying cortisol secretion, and through a simulation study we show that our formulation can control the cortisol levels to remain within the desired bounds while having the circadian and the ultradian rhythms underlying cortisol secretion. Hence, while our approach uses control-theoretic concepts to understand a biological process, the proposed formulation is a first step in developing intermittent controllers for curing cortisol deficiency. While the methods proposed in our study are not externally applied to control a biological process, with slight modifications based on the pathological condition of interest, the proposed intermittent controller can be used to control some of the pathological problems related to cortisol. This can be done by including the first-order kinetics of the medicine that will be injected to the patient to control cortisol levels, and then using compressed sensing algorithms to recover the secretory release of cortisol in the patient. In this case there will be two sets of pulses that control cortisol levels: (i) external pulses that are injected to the patient (ii) pulses that are secreted as a part of the natural control system underlying cortisol secretion. Similarly, such bio-inspired controllers can be used for controlling other hormones (e.g., growth hormone, thyroid hormone, or gonadal hormones).

Since this type of controllers can be adapted to be applied for curing different pathological conditions related to endocrine hormones, the idea behind modeling such controllers opens new research directions. For example, a patient who suffers from Addisons disease takes cortisone once or twice a day for their cortisol deficiency (which does not seem optimal), while an impulse controller can be used to control the cortisol levels optimally. The future directions of this research include designing an impulse controller such that the optimality of the controller is guaranteed. Moreover, in brain-machine interface design, in which brain implants control epilepsy or Parkinson's disease, it is possible to design pulse controllers instead of continuous controllers to improve the battery life of the brain implant and reduce the number of surgeries required for changing the battery of the implanted controller. With the new advances and ongoing research in brain-machine interface design for psychiatric disorders, this type of pulse controller can potentially be used to control post-traumatic stress disorder, major depression, and addiction. For example, in post-traumatic stress disorder or major depression, in theory, one could potentially measure skin conductance response which results from discrete emotional shocks experienced by the patient, and ideally stimulate ventromedial prefrontal cortex using impulse control to reverse the effect of the emotional shocks in the patient. In conclusion, inspired by the pulse controller proposed in this research, it is potentially possible to design a class of pulse controllers for applications that naturally arise in neuroscience.

## Author contributions

RTF, MAD, and ENB designed the optimal control formulation. RTF performed research and wrote the paper.

## Funding

RTF's work was supported in part by the NSF Graduate Fellowship. For this work, ENB is supported in part by NIH DP1 OD003646, 1-R01-GM104948-03 and NSF 0836720, and MAD is supported in part by EFRI-0735956. The funders had no role in study design and analysis, decision to publish, or preparation of the manuscript.

### Conflict of interest statement

The authors declare that the research was conducted in the absence of any commercial or financial relationships that could be construed as a potential conflict of interest.
